# Draft Genome Sequence of Pseudomonas citronellolis LA18T, a Bacterium That Uses Levulinic Acid

**DOI:** 10.1128/MRA.00906-18

**Published:** 2018-08-09

**Authors:** Tomohiro Inaba, Yuya Sato, Hideaki Koike, Tomoyuki Hori, Manabu Kanno, Nobutada Kimura, Kohtaro Kirimura, Hiroshi Habe

**Affiliations:** a Environmental Management Research Institute, National Institute of Advanced Industrial Science and Technology, Tsukuba, Ibaraki, Japan; b Bioproduction Research Institute, National Institute of Advanced Industrial Science and Technology, Tsukuba, Ibaraki, Japan; c Department of Applied Chemistry, Faculty of Science and Engineering, Waseda University, Shinjuku, Tokyo, Japan; University of Arizona

## Abstract

Pseudomonas citronellolis LA18T catabolizes levulinic acid (LA) from cellulosic biomass hydrolysate via acetyl-coenzyme A (acetyl-CoA) and propionyl-CoA. This study reports the 7.22-Mbp draft genome sequence of P. citronellolis LA18T. The draft genome sequence will aid the study of the LA catabolic pathway, which will allow for more applications of LA-utilizing bacteria.

## Abstract (precis)

Pseudomonas citronellolis LA18T catabolizes levulinic acid (LA) from cellulosic biomass hydrolysate via acetyl-coenzyme A (acetyl-CoA) and propionyl-CoA. This study reports the 7.22-Mbp draft genome sequence of P. citronellolis LA18T.

## ANNOUNCEMENT

Levulinic acid (LA) is a C_5_ γ-keto acid that can be obtained from biomass resources and is a promising building block for biobased chemical products (1, 2). The recent discovery of a unique operon responsible for LA catabolism in Pseudomonas putida KT2440 has led to an interest in more efficient utilization of LA (3). Previously, we isolated several LA-utilizing bacteria, including Pseudomonas sp. strain LA18T, and identified several metabolites during LA catabolism (4, 5). Strain LA18T can utilize not only reagent-grade LA but also cedar-derived LA via acetic acid and propionic acid as intermediates (4, 6, 7); however, the molecular basis of LA catabolism in LA18T remains unclear.

Strain LA18T was isolated from a soil sample, as previously reported (4). The single colony of LA18T was picked up and cultured for preparation of a whole-genome sample. Total DNA was extracted from cultured suspension using DNeasy blood and tissue kits (Qiagen, Hilden, Germany) according to the manufacturer’s instructions. The draft genome sequence of LA18T was generated with the MiSeq next-generation sequencing platform (Illumina, San Diego, CA). Both paired-end and mate pair DNA libraries (insert size, ∼500 bp) were prepared using a NEBNext Ultra DNA library prep kit for Illumina (New England BioLabs, Ipswich, MA) and a Nextera mate pair sample prep kit (Illumina), respectively, and sequenced on a MiSeq platform using the MiSeq reagent kit version 2 (Illumina). Sequence data totaling 3.13 million paired-end and 0.12 million mate pair reads, each approximately 250 and 2,000 bp in length, respectively, were generated. Genomic sequence assembly using ALLPATHS-LG version 46449 (8) with default settings generated 29 scaffolds composed of 179 contigs and a 7.22-Mb draft genome sequence at 100-fold coverage from both the paired-end and mate pair libraries. The length of the longest scaffold was 848,153 bp, and the *N*_50_ length was 3,751,832 bp, with six scaffolds. A total of 6,360 protein-coding genes were predicted using Glimmer 3.02 (9), with default settings, and annotated using NCBI blast 2.2.29 (blastp) with RefSeq version 65 (10, 11). The cutoff values used for gene annotation were an E value of ≤1e-10 and ≥25% amino acid sequence identity. A total of 60 tRNA- and 9 rRNA-encoding genes were also identified by tRNAscan-SE 1.3.1, with the general tRNA model option and RNAmmer 1.2, with default settings, respectively (12, 13). The 16S rRNA gene sequence of LA18T shared >99.3% identity with that of Pseudomonas citronellolis P3B5. (14). To date, no possible LA catabolism gene operon similar to that in P. putida KT2440 has been found in the LA18T genome. Hence, the transcriptome analysis based on the draft genome sequence may provide some novel information on LA catabolism genes in Pseudomonas species.

### Data availability.

The P. citronellolis LA18T genome sequence has been deposited as 179 contigs and 29 scaffolds (accession numbers BGPP01000001 to BGPP01000029) in DDBJ/EMBL/GenBank. The version described in this paper is the first version.
